# The *Nazeris* Fauna of the Luoxiao Mountain Range, China. (Coleoptera, Staphylinidae, Paederinae)

**DOI:** 10.1371/journal.pone.0131942

**Published:** 2015-07-10

**Authors:** Jia-Yao Hu, Li-Zhen Li

**Affiliations:** Department of Biology, College of Life and Environmental Sciences, Shanghai Normal University, 100 Guilin Road, Shanghai, 200234 P. R. China; Roehampton University, UNITED KINGDOM

## Abstract

Fourteen species of *Nazeris* Fauvel are recorded from the Luoxiao Mountains. Twelve of them are described as new: *N*. *luoxiaoshanus*, *N*. *pengzhongi*, *N*. *divisus*, *N*. *paradivisus*, *N*. *xiaobini*, *N*. *congchaoi*, *N*. *nannani*, *N*. *rufus*, *N*. *ziweii*, *N*. *daweishanus*, *N*. *prominens*, and *N*. *zekani*. *N*. *inaequalis* Assing, 2014 is newly recorded from Hunan Province.

## Introduction

The speciose paederine genus *Nazeris* Fauvel previously included 233 named species and seven subspecies [1]. The genus is distinguished from other paederines particularly by the morphology of the aedeagus, which has a pair of dorso-lateral apophyses [2].

Up to the present, 139 species and one subspecies have been recorded from China. Koch described the first two species, *N*. *chinensis* and *N*. *minor* from Zhejiang [3]. Ito added 19 species and one subspecies from Taiwan [4–9], and one species from Zhejiang [10]. Zheng described four species from Sichuan [11]. Li (1993) added one species from Anhui in the genus *Scopaeus* Erichson [12], which was transferred to *Nazeris* by Frisch [13]. Watanabe *et al* recorded nine species from Yunnan [14–16]. Hu *et al*. added 37 species mainly from Zhejiang, Guangxi and Sichuan [17–27]. Assing added 66 species primarily from Yunnan, Sichuan, Gansu and Shaanxi [28–30].

The Luoxiao Mountains range from central to eastern China, extending for about 400 km through Hubei, Hunan and Jiangxi provinces, with several peaks of more than 2000 m. Only two *Nazeris* species were previously known from this large area: *N*. *inaequalis* Assing and *N*. *proiectus* Assing. During several recent field trips to this region conducted by the authors and their colleagues, the two known species were collected at their type localities and nearby areas. Besides, twelve undescribed species were discovered. In the present paper, we describe the new species and provide illustrations of their major diagnostic features.

## Material and Methods

This study is part of a joint investigation project of biodiversity in the Luoxiao Mountain Range. Apermit was provided by the ministry of Science and Technology of China. The field studies did not involve endangered or protected species.

The type material listed in the present study is deposited in the Insect Collection of Shanghai Normal University, Shanghai, P. R. China (SNUC).

The dissected body parts were mounted in Euparal on plastic slides. The habitus photos were taken using a Canon 7D camera. The photos of the sternites and aedeagi were taken using a Canon G9 camera mounted on an Olympus CX31 microscope.

### Measurements

Body length: measured from the anterior margin of the labrum to the apex of the abdomen.
Length of forebody: measured from anterior margin of the labrum to the posterior margin of the elytra.
Eye length: longitudinal length of eye in dorsal view.
Postocular length: measured from posterior margin of eye to posterior constriction of head.
Head width: width of head across (and including) eyes.
Head length: measured from the clypeal anterior margin to head base.
Pronotum width: maximal width of pronotum.
Pronotum length: measured in midline from front margin to posterior margin.
Width of elytra: combined width of elytra at posterior margin.
Length of elytra: measured from apex of scutellum to posterior margin.

### Nomenclatural Acts

The electronic edition of this article conforms to the requirements of the amended International Code of Zoological Nomenclature, and hence the new names contained herein are available under that Code from the electronic edition of this article. This published work and the nomenclatural acts it contains have been registered in ZooBank, the online registration system for the ICZN. The ZooBank LSIDs (Life Science Identifiers) can be resolved and the associated information viewed through any standard web browser by appending the LSID to the prefix "http://zoobank.org/". The LSID for this publication is: urn:lsid:zoobank.org:pub:2F1A7824-2D0C-44E5-99AE-E4ECB4A3CA36. The electronic edition of this work was published in a journal with an ISSN, and has been archived and is available from the following digital repositories: PubMed Central, LOCKSS.

## Results and Discussion

### Additional records

#### *Nazeris inaequalis* Assing, 2014

*Material examined*. CHINA: Western Jiangxi: 1 male, 2 females, Ji'an City, Jinggang Shan, Bijia Shan, 26°30′19″N, 114°09′25″E, mixed leaf litter, sifted, 1330 m, 25.VII.2014, Chen, Hu, Lv & Yu leg.; 3 males, 2 females, Luxi County, Yangshimu, 27°35′07″N, 114°15′41″E, mixed forest, leaf litter, wood, sifted & beating, ca. 1360 m, 24.X.2013, Peng, Shen & Yan leg.; 1 female, Yichun City, Mingyue Shan, 27°35′13″N, 114°16′53″E, mixed forest, leaf litter, wood, sifted & beating, ca. 1600 m, 22.X.2013, Peng, Shen & Yan leg.; 1 female, Yichun City, Mingyue Shan, 27°35′44″N, 114°16′26″E, mixed forest, leaf litter, wood, sifted & beating, ca. 1140 m, 23.X.2013, Peng, Shen & Yan leg. Eastern Hunan: 1 male, Yanling County, Taoyuandong National Park, 26°29′55″N, 114°02′54″E, along path in mixed forest, leaf litter, sifted, 900 m, 17.VII.2013, Dai, Peng, Xie leg.; 1 female, Yanling County, Taoyuandong National Park, 26°29′44″N, 114°04′39″E, along path in mixed forest, leaf litter, sifted, 1200 m, 18.VII.2013, Dai, Peng, Xie leg.

*Distribution and habitat data*. The species was found in several localities in the central and southern parts of Luoxiao Shan in western Jiangxi and eastern Hunan (new province record). The specimens were collected by sifting leaf litter in mixed forests, partly together with *N*. *luoxiaoshanus*, *N*. *pengzhongi*, or *N*. *prominens*. The altitudes range from 900 to 1600 m.

*Remarks*. The male sexual characters of the above material are identical to those illustrated by Assing (2014: figs 53–59) [30].

#### *Nazeris proiectus* Assing, 2014

*Material examined*. CHINA: Western Jiangxi: 3 males, 3 females, Ji'an City, Jinggang Shan, Bijia Shan, 26°31′08″N, 114°11′22″E, mixed leaf litter, sifted, 400–600 m, 23.VII.2014, Chen, Hu, Lv & Yu leg.; 1 female, Ji'an City, Jinggang Shan, Bijia Shan, 26°31′03″N, 114°11′17″E, mixed leaf litter, sifted, 580 m, 24.VII.2014, Chen, Hu, Lv & Yu leg.

*Distribution and habitat data*. The species was found only in Jinggang Shan in the southern part of Luoxiao Shan in western Jiangxi. The specimens were collected by sifting leaf litter in mixed forests at altitudes between 400 and 600 m.

*Remarks*. The species is similar to *N*. *shenshanjiai* Hu et al., 2011, from southern Zhejiang province in general appearance and aedeagal characters [24], but can be separated by the abdominal tergites lacking microsculpture, by the posterior margin of male sternite VII having a median projection, and by the dorso-lateral apophyses extending beyond the apex of the ventral process. The sexual characters of the above males are identical to those illustrated by Assing (2014: figs 60–66) [30].

### Descriptions of new species

#### *Nazeris luoxiaoshanus* Hu & Li sp. n.

urn:lsid:zoobank.org:act:027FCC7B-612E-47D5-B569-F8B42933E866

*Type material*. Holotype: male: ‘China: W. Jiangxi, Yichun City, Mingyueshan National Park, 27°35′43–41″N, 114°16′29–05″E, along path in mixed forest, bamboo, leaf litter, sifted, 700–1150 m, 13.VII.2013, Song, Yin, Yu leg.’ (SNUC). Paratypes: 7 males, 10 females: same data as holotype; 1 male, 3 females: ‘China: W. Jiangxi, Yichun City, Mingyueshan National Park, 27°35′05″N, 114°17′34″E, along plank road, broad-leaved forest, sifted, ca. 1420 m, 11.VII.2013, Song, Yin, Yu leg.’; 1 male: ‘China: Jiangxi, Pingxiang City, Luxi County, Yangjia Ling, 27°35′03″N, 114°15′02″E, path in leaf litter, sifted, ca. 820 m, bamboo forest, wood with fungi, 15.VII.2013, Song, Yin, Yu leg.’; 1 male, 3 females: ‘China: Jiangxi, Pingxiang City, Luxi County, Yang-shi-mu Area, 27°34′25″–33′38″N, 114°14′14″–28″E, broad-leaved forest, leaf, bamboo litter, sifted, 910–1550 m, 16.VII.2013, Song, Yin, Yu leg.’; 8 males, 13 females: ‘China: Jiangxi, Pingxiang City, Wugong Shan National Park, 27°27′39″N, 114°10′03″E, cableway station to Dian-jiang-tai, broad leaf, sifted, 1340–1400 m, 19.VII.2013, Song, Yin, Yu leg.’; 9 males, 4 females: ‘China: Jiangxi, Pingxiang City, Wugong Shan National Park, 27°27′55″N, 114°09′58″E, cableway station to Biaoshui Waterfall, broad leaf, sifted, 1000–1350 m, 20.VII.2013, Song, Yin, Yu’; 4 males, 3 females: ‘China: Jiangxi, Pingxiang City, Wugong Shan National Park, 27°27′26″N, 114°10′12″E, cableway station to Huiyin Gu, bamboo & pine leaf, sifted, 1500–1750 m, 21.VII.2013, Song, Yin, Yu’; 1 male: ‘China: W. Jiangxi Province, Yichun City, Mingyue Shan, 27°35′13″N, 114°16′53″E, mixed forest, leaf litter, wood, sifted & beating, ca. 1600 m, 22.X.2013, Peng, Shen & Yan leg.’; 9 males, 12 females: ‘China: W. Jiangxi Province, Yichun City, Mingyue Shan, 27°35′44″N, 114°16′26″E, mixed forest, leaf litter, wood, sifted & beating, ca. 1140 m, 23.X.2013, Peng, Shen & Yan leg.’; 10 males, 8 females: ‘China: W. Jiangxi Province, Luxi County, Yangshimu, 27°35′07″N, 114°15′41″E, mixed forest, leaf litter, wood, sifted & beating, ca. 1360 m, 24.X.2013, Peng, Shen & Yan’; 1 male, 2 females: ‘China: W. Jiangxi Province, Luxi County, Wugong Shan, 27°27′53″N, 114°10′47″E, mixed forest, leaf litter, wood, sifted & beating, ca. 1570 m, 27.X.2013, Peng, Shen & Yan’; 2 females: ‘China: W. Jiangxi Province, Luxi County, Wugong Shan, 27°27′59″N, 114°09′54″E, mixed forest, leaf litter, wood, sifted & beating, ca. 1050 m, 28.X.2013, Peng, Shen & Yan’.

*Description*. Body length 3.8–4.6 mm; length of forebody 2.4–2.5 mm.

**Fig 1:**
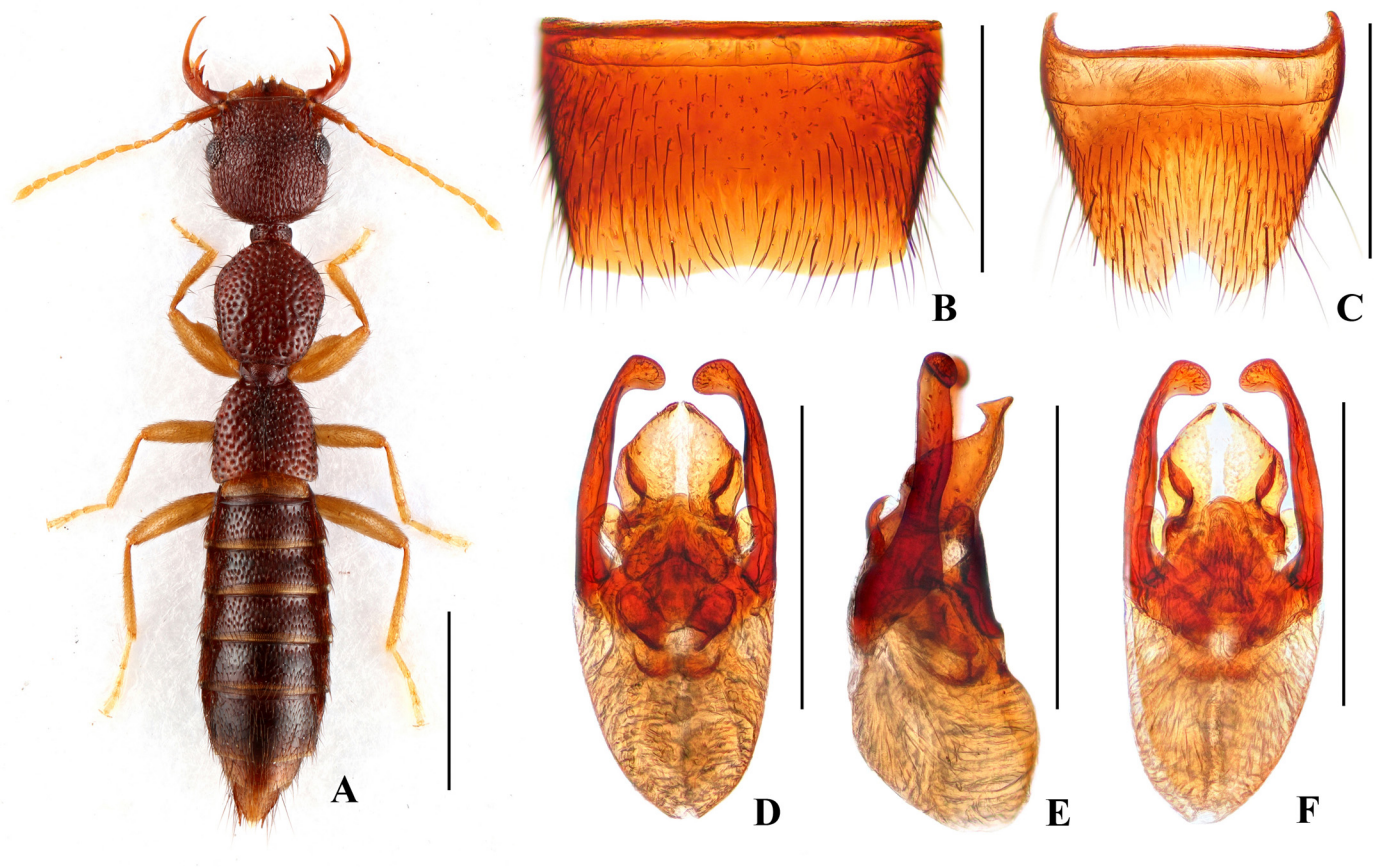
*Nazeris luoxiaoshanus*. (A) habitus. (B) male sternite VII. (C) male sternite VIII. (D) aedeagus, in ventral view. (E) aedeagus, in lateral view. (F) aedeagus, in dorsal view. Scales: A = 1 mm, B–F = 0.5 mm.

Forebody (Fig 1A) reddish brown; abdomen somewhat darker; antennae and legs yellow.

Head as long as wide; punctation very dense, rather coarse, partly confluent, and distinctly umbilicate, interstices without microsculpture; postocular portion 1.68 times as long as eye length.

Pronotum 1.13 times as long as wide, 0.89 times as broad and as long as head; punctation dense and much coarser than that of head; midline with narrow impunctate elevation in posterior half; interstices without microsculpture. Elytra 0.68 times as long as wide, 0.59 times as long and 0.97 times as broad as pronotum; punctation as coarse and as dense as that of pronotum; interstices without microsculpture.

Abdomen with punctation dense and coarse on tergites III–V, less dense and finer on tergite VI, sparse and fine on tergites VII–VIII; interstices lacking microsculpture.

*Male*. Sternite VII (Fig 1B) with posterior margin shallowly concave in the middle. Sternite VIII (Fig 1C) with triangular excision posteriorly. Aedeagus (Fig 1D–1F) weakly sclerotized; ventral process short and wide, with pair of small basal laminae ventrally, apex with dorsal process in lateral view; dorso-lateral apophyses moderately slender, apically bent mediad in ventral view, extending beyond apex of ventral process.

*Distribution and habitat data*. The species was found in several localities in the central part of Luoxiao Shan in western Jiangxi. The specimens were collected by sifting decaying leaf litter in mixed forests and bamboo forests, partly together with *N*. *ziweii*, *N*. *prominens*, or *N*. *inaequalis*. The altitudes range from 700 to 1750 m. Several paratypes are teneral.

*Remarks*. The new species is similar to *N*. *luoi* Hu & Li, [26] and *N*. *tani* Hu & Li, [26], but can be separated by the different aedeagal structure, especially the shapes of the ventral process and the dorso-lateral apophyses.

*Etymology*. The specific epithet is derived from Luoxiao Shan, where the species was discovered.

#### *Nazeris pengzhongi* Hu & Li sp. n.

urn:lsid:zoobank.org:act:14BA1781-A980-4CFC-BF2A-CFC9F69CD00F

*Type material*. Holotype: male: ‘China: Hunan, Yanling County, Taoyuandong National Park, 26°29′44″N, 114°04′39″E, along path in mixed forest, leaf litter, sifted, 1200 m, 18.VII.2013, Dai, Peng, Xie leg.’ (SNUC). Paratypes: 2 males, 3 females: same data as holotype; 1 male, 3 females: ‘China: Hunan, Yanling County, Taoyuandong National Park, 26°29′14″N, 114°′42″E, along path in mixed forest, leaf litter, sifted, 770 m, 16.VII.2013, Dai, Peng, Xie leg.’; 3 males, 1 female: China: Jiangxi Prov. Jinggangshan City, Ciping Town, Jingzhushan, alt. 1000–1200 m, 19.X.2010, Peng, Zhai & Zhu leg.’; 6 males, 14 females: ‘China: W. Jiangxi, Ji'an City, Jinggang Shan, Huangyangjie, 26°37′25″N, 114°06′58″E, *Cunninghamia lanceolata* leaf litter, sifted, 1240 m, 28.VII.2014, Chen, Hu, Lv & Yu leg.’; 2 males, 8 females: ‘China: W. Jiangxi, Ji'an City, Jinggang Shan, Longtan, 26°35′47″N, 114°08′25″E, mixed leaf litter, sifted, 760–820 m, 29.VII.2014, Chen, Hu, Lv & Yu leg.’; 11 males, 7 females: ‘China: W. Jiangxi, Ji'an City, Jinggang Shan, Shuikou, 26°32′42″N, 114°08′03″E, mixed leaf litter, sifted, 790–900 m, 30.VII.2014, Chen, Hu, Lv & Yu leg.’; 39 males, 21 females: ‘China: W. Jiangxi, Ji'an City, Jinggang Shan, Jingzhu Shan, 26°29′45″N, 114°04′45″E, mixed leaf litter, sifted, 1160 m, 31.VII.2014, Chen, Hu, Lv & Yu leg.’; 15 males, 12 females: ‘China: W. Jiangxi, Ji'an City, Jinggang Shan, Jingzhu Shan, 26°29′45″N, 114°04′45″E, mixed leaf litter, sifted, 1160 m, 2.VIII.2014, Chen, Hu, Lv & Yu leg.’.

*Description*. Body length 4.2–4.5 mm; length of forebody 2.7–2.9 mm.

**Fig 2:**
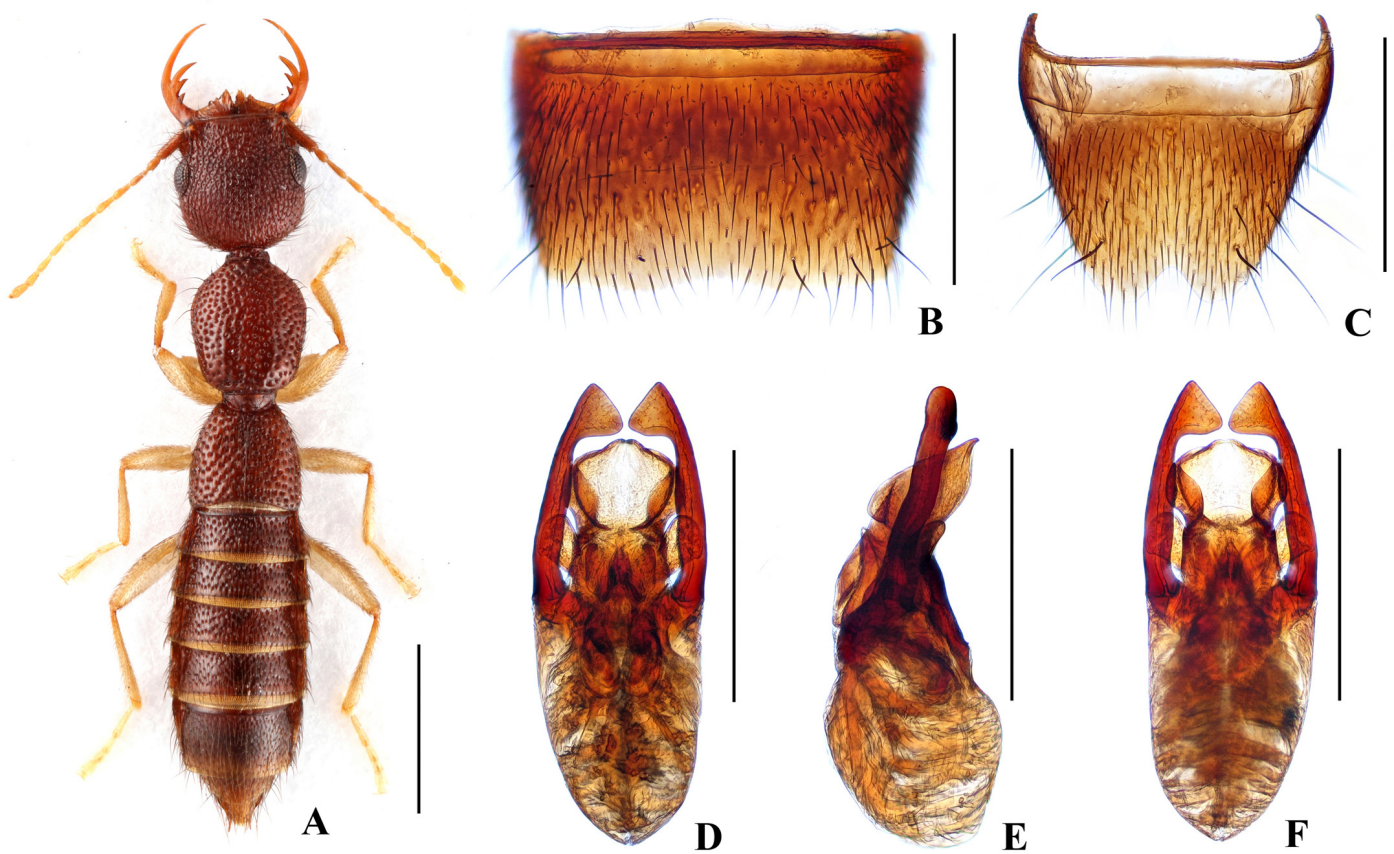
Nazeris pengzhongi. (A) habitus. (B) male sternite VII. (C) male sternite VIII. (D) aedeagus, in ventral view. (E) aedeagus, in lateral view. (F) aedeagus, in dorsal view. Scales: A = 1 mm, B–F = 0.5 mm.

Forebody (Fig 2A) reddish brown; abdomen somewhat darker; antennae and legs yellow.

Head as long as wide; punctation very dense, rather coarse, partly confluent, and distinctly umbilicate, interstices without microsculpture; postocular portion 1.45 times as long as eye length.

Pronotum 1.17 times as long as wide, 0.87 times as broad and as long as head; punctation dense and much coarser than that of head; midline with narrow impunctate elevation in posterior half; interstices without microsculpture. Elytra 0.65 times as long as wide, 0.55 times as long and 0.97 times as broad as pronotum; punctation as coarse and as dense as that of pronotum; interstices without microsculpture.

Abdomen with punctation dense and coarse on tergites III–V, less dense and finer on tergite VI, sparse and fine on tergites VII–VIII; interstices lacking microsculpture.

*Male*. Sternite VII (Fig 2B) with posterior margin weakly concave in the middle. Sternite VIII (Fig 2C) with broadly triangular posterior excision. Aedeagus (Fig 2D–2F) weakly sclerotized; ventral process short and wide, with pair of basal laminae ventrally; dorso-lateral apophyses slender, triangularly widened at apex in ventral view, nearly straight in lateral view, extending beyond apex of ventral process.

*Distribution and habitat data*. The species was found in several localities in the southern part of Luoxiao Shan in eastern Hunan and in western Jiangxi. The specimens were collected by sifting decaying leaf litter in mixed forests at altitudes of 760–1240 m, partly together with *N*. *inaequalis* or *N*. *congchaoi*. Several paratypes are teneral.

*Remarks*. The new species is similar to *N*. *luoxiaoshanus* in general appearance and aedeagal characters, but can be separated by the shapes of the ventral process of the aedeagus (apex simply acute in lateral view) and of the dorso-lateral apophyses (triangularly widened at apex in ventral view).

*Etymology*. The species is named in honor of Zhong Peng, who collected some type material of the new species.

#### *Nazeris divisus* Hu & Li sp. n.

urn:lsid:zoobank.org:act:A4467DEB-ADBC-41EC-B66B-B0B6AD596787

*Type material*. Holotype: male: ‘China: Hunan, Liuyang City, Daweishan National Park, 28°25′28″N, 114°04′52″E, along path in mixed forest, leaf litter, sifted, 830 m, 22.VII.2013, Dai, Peng, Xie leg.’ (SNUC). Paratypes: 1 male: ‘China: Hunan, Liuyang City, Daweishan National Park, 28°25′43″N, 114°08′56″E, along path in mixed forest, leaf litter, sifted, 1450 m, 20.VII.2013, Dai, Peng, Xie leg.’; 4 females: ‘China: Hunan, Liuyang City, Daweishan National Park, 28°25′37″N, 114°07′43″E, along path in mixed forest, leaf litter, sifted, 1430 m, 21.VII.2013, Dai, Peng, Xie leg.’.

*Description*. Body length 5.2–5.8 mm; length of forebody 2.9–3.1 mm.

**Fig 3:**
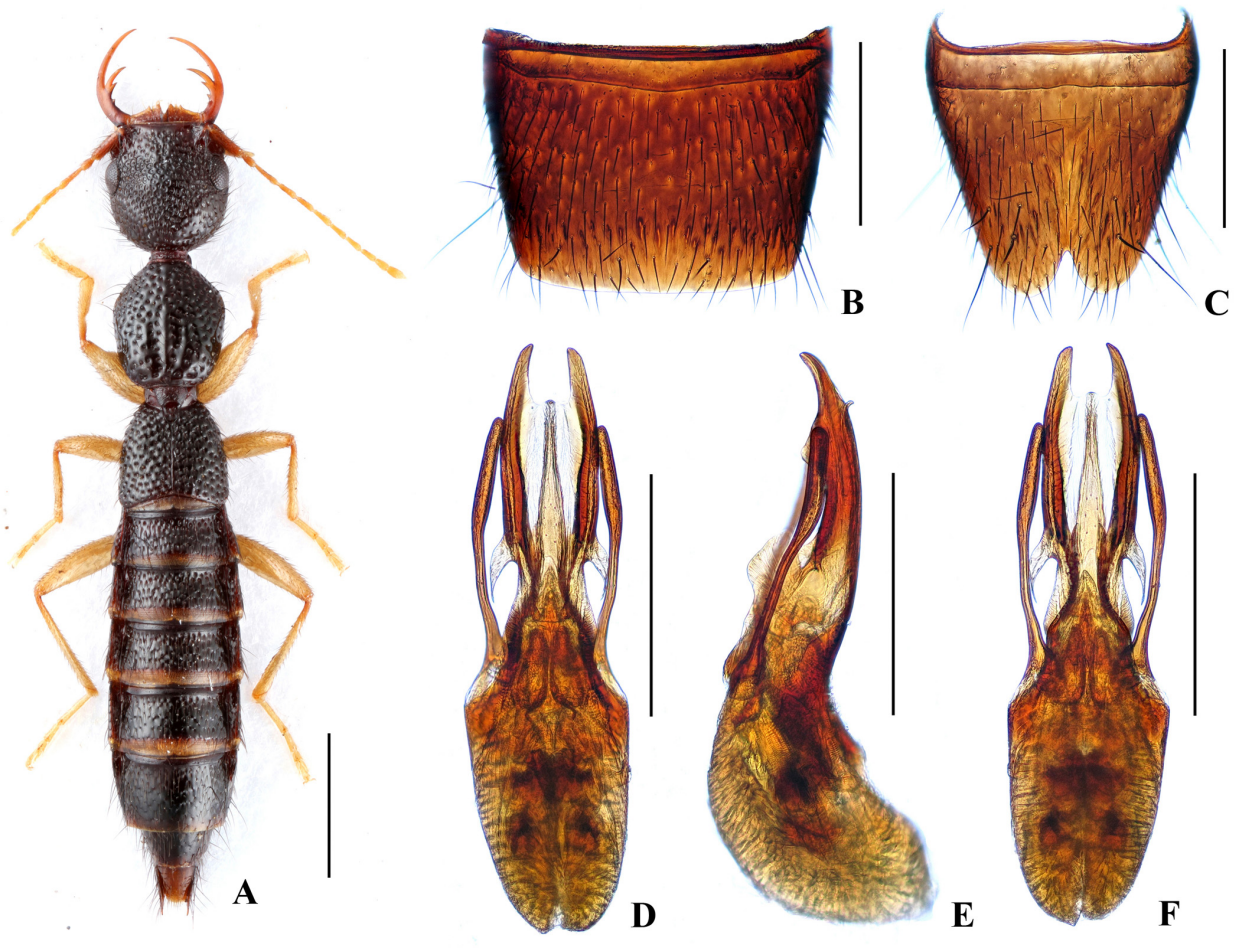
Nazeris divisus. (A) habitus. (B) male sternite VII. (C) male sternite VIII. (D) aedeagus, in ventral view. (E) aedeagus, in lateral view. (F) aedeagus, in dorsal view. Scales: A = 1 mm, B–F = 0.5 mm.

Body (Fig 3A) dark brown; antennae and legs yellow.

Head as long as wide; punctation very dense, rather coarse, not confluent, and distinctly umbilicate, interstices without microsculpture; postocular portion 1.53 times as long as eye length.

Pronotum 1.13 times as long as wide, 0.89 times as broad and as long as head; punctation moderately dense and much coarser than that of head; midline with narrow impunctate elevation in posterior half; lateral portions with irregular longitudinal glossy callosities; interstices without microsculpture. Elytra 0.64 times as long as wide, 0.56 times as long and as broad as pronotum; punctation denser but less coarse than that of pronotum; interstices without microsculpture.

Abdomen with punctation dense and coarse on tergites III–V, less dense and finer on tergite VI, sparse and fine on tergites VII–VIII; interstices lacking microsculpture.

*Male*. Sternite VII (Fig 3B) with posterior margin truncate at middle. Sternite VIII (Fig 3C) with acutely V-shaped posterior excision. Aedeagus (Fig 3D–3F) well sclerotized; ventral process long, constricted in basal third, with pair of small basal laminae ventrally, apex divided into two straight branches in ventral view; dorso-lateral apophyses slender, slightly curved in ventral view, not reaching apex of ventral process.

*Distribution and habitat data*. The species is known only from Dawei Shan in northeastern Hunan. The specimens were collected by sifting decaying leaf litter in mixed forests at altitudes of 830–1450 m, partly together with *N*. *nannani*, *N*. *zekani* or *N*. *daweishanus*. One paratype is teneral.

*Remarks*. The new species is similar to *N*. *grandis* Hu & Li, [26] from eastern Guangxi province in external characters and male sternites, but can be separated by smaller body size and the different aedeagal structure.

*Etymology*. The specific epithet is the past participle of the Latin verb dividere (to divide), alluding to the apically divided ventral process of aedeagus.

#### *Nazeris paradivisus* Hu & Li sp. n.

urn:lsid:zoobank.org:act:21DB5E6B-03A3-457B-A941-2F26AAAD78B3

*Type material*. Holotype: male: ‘China: W. Jiangxi, Yichun City, Fengxin County, Baizhang Shan, 28°42′40″N, 114°46′35″E, *Cunninghamia lanceolata* leaf litter, sifted, 800–1100 m, 17.VII.2013, Hu & Lv leg.’ (SNUC). Paratypes: 8 males, 6 females: same data as holotype; 1 male: ‘China: W. Jiangxi, Yichun City, Fengxin County, Baizhang Vill., 28°41′35″N, 114°46′27″E, mixed leaf litter, sifted, 800 m, 14.VII.2013, Hu & Lv leg.’; 2 males, 1 female: ‘China: W. Jiangxi, Yichun City, Fengxin County, Baizhang Vill., 28°41′18″N, 114°46′13″E, mixed leaf litter, sifted, 840–860 m, 15.VII.2013, J. Y. Hu & Z. K. Lv leg.’; 5 males, 2 females: ‘China: W. Jiangxi, Yichun City, Fengxin County, Jiuling Shan, 28°41′57″N, 114°44′33″E, pine leaf litter, sifted, 1250 m, 19.VII.2013, Hu & Lv leg.’; 6 males, 9 females: ‘China: W. Jiangxi, Yichun City, Fengxin County, Niyang Shan, 28°49′12″N, 115°03′26″E, mixed leaf litter, sifted, 1000 m, 24.VII.2013, Hu & Lv leg.’.

*Description*. Body length 5.3–6.1 mm; length of forebody 2.9–3.1 mm.

**Fig 4:**
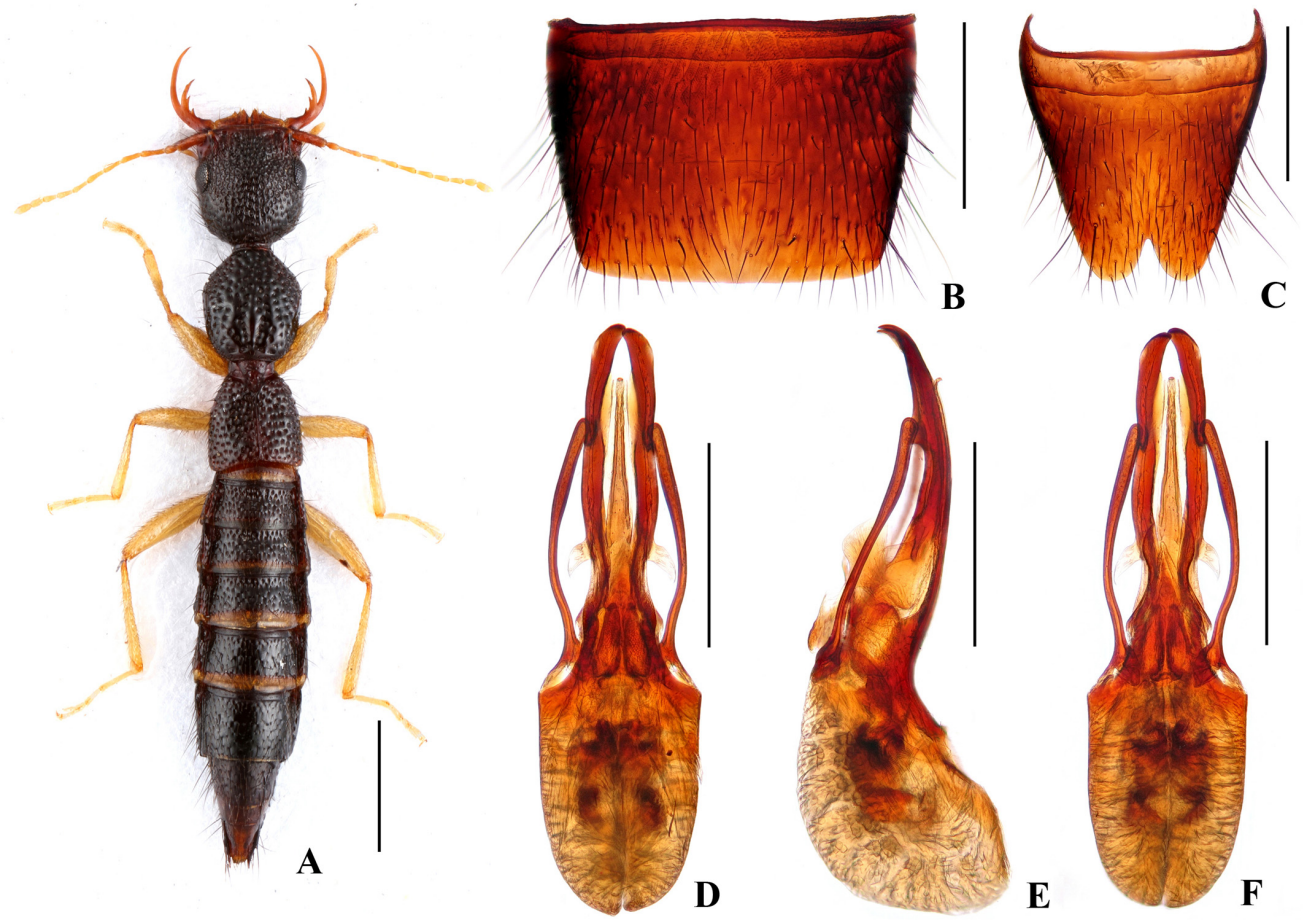
Nazeris paradivisus. (A) habitus. (B) male sternite VII. (C) male sternite VIII. (D) aedeagus, in ventral view. (E) aedeagus, in lateral view. (F) aedeagus, in dorsal view. Scales: A = 1 mm, B–F = 0.5 mm.

Body (Fig 4A) dark brown; antennae and legs yellowish brown.

Head as long as wide; punctation very dense, rather coarse, not confluent, and distinctly umbilicate, interstices without microsculpture; postocular portion 1.52 times as long as eye length.

Pronotum 1.16 times as long as wide, 0.89 times as broad and as long as head; punctation moderately dense and much coarser than that of head; midline with narrow impunctate elevation in posterior half; lateral portions with irregular longitudinal glossy callosities; interstices without microsculpture. Elytra 0.69 times as long as wide, 0.56 times as long and 0.95 times as broad as pronotum; punctation denser but less coarse than that of pronotum; interstices without microsculpture.

Abdomen with punctation dense and coarse on tergites III–V, less dense and finer on tergite VI, sparse and fine on tergites VII–VIII; interstices lacking microsculpture.

*Male*. Sternite VII (Fig 4B) with posterior margin truncate at middle. Sternite VIII (Fig 4C) with V-shaped posterior excision. Aedeagus (Fig 4D–4F) well sclerotized; ventral process long, constricted in basal third, with pair of small basal laminae ventrally, apex divided into two curved branches in ventral view; dorso-lateral apophyses slender, slightly curved in ventral view, not reaching apex of ventral process.

*Distribution and habitat data*. The species was found in three adjacent localities to the north of Yichun City, in northwestern Jiangxi. The specimens were collected by sifting *Cunninghamia lanceolata*, pine or mixed leaf litter at altitudes of 800–1250 m. Several paratypes are teneral.

*Remarks*. The new species is similar to *N*. *divisus* in general appearance and aedeagal characters, but can be separated by the narrower ventral process with wider and curved apical processes.

*Etymology*. The species epithet (adjective) refers to the similarity of this species to *N*. *divisus*.

#### *Nazeris xiaobini* Hu & Li sp. n.

urn:lsid:zoobank.org:act:75576AC9-F9D4-4ED6-A13E-0FAF265C302F

*Type material*. Holotype: male: ‘China: Jiangxi, Pingxiang City, Gaozhou County, Gao-tian-yan, 27°23′51″N, 114°00′54″E, broad-leaved & pine forest, mixed litter, sifted, ca. 1025 m, 23.VII.2013, Song, Yin, Yu leg.’ (SNUC). Paratypes: 2 males, 2 females: same data as holotype.

*Description*. Body length 5.1–5.8 mm; length of forebody 2.7–3.0 mm.

**Fig 5:**
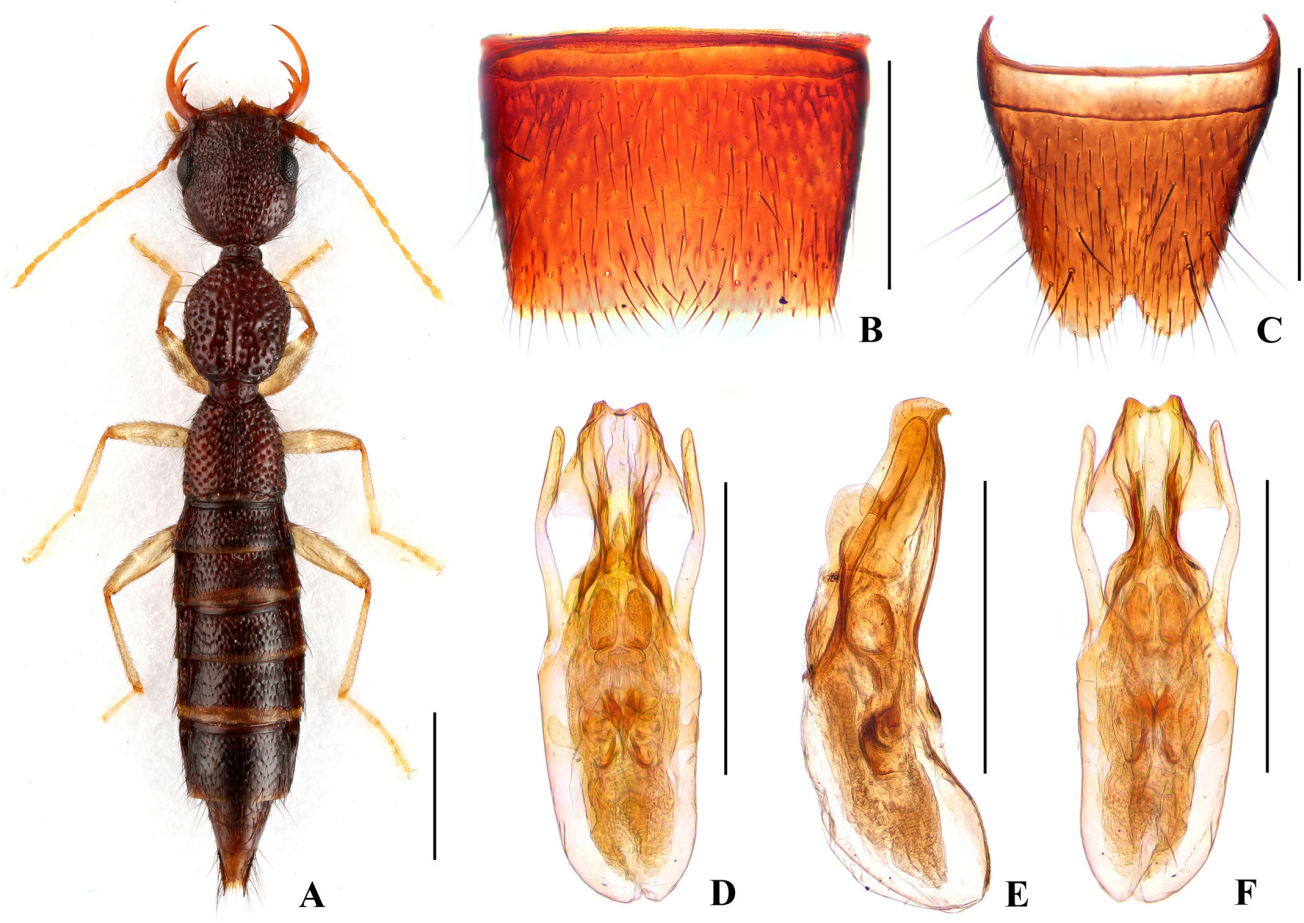
Nazeris xiaobini. (A) habitus. (B) male sternite VII. (C) male sternite VIII. (D) aedeagus, in ventral view. (E) aedeagus, in lateral view. (F) aedeagus, in dorsal view. Scales: A = 1 mm, B–F = 0.5 mm.

Body (Fig 5A) dark brown, somewhat reddish; antennae and legs yellow.

Head longer than wide (length/width = 1.04); punctation very dense, rather coarse, not confluent, and distinctly umbilicate, interstices without microsculpture; postocular portion 1.45 times as long as eye length.

Pronotum 1.18 times as long as wide, 0.88 times as broad and as long as head; punctation moderately dense and much coarser than that of head; midline with narrow impunctate elevation in posterior half; lateral portions with irregular longitudinal glossy callosities; interstices without microsculpture. Elytra 0.67 times as long as wide, 0.55 times as long and 0.96 times as broad as pronotum; punctation denser but less coarse than that of pronotum; interstices without microsculpture.

Abdomen with punctation dense and coarse on tergites III–V, less dense and finer on tergite VI, sparse and fine on tergites VII–VIII; interstices lacking microsculpture.

*Male*. Sternite VII (Fig 5B) with posterior margin truncate at middle. Sternite VIII (Fig 5C) with broadly V-shaped posterior excision. Aedeagus (Fig 5D–5F) weakly sclerotized; ventral process triangularly widened in apical half and shallowly concave at apex in ventral view; dorso-lateral apophyses slender, slightly curved in ventral view, not reaching apex of ventral process.

*Distribution and habitat data*. The type locality is situated to the south of Pingxiang City, in western Jiangxi. The specimens were collected by sifting decaying leaf litter in mixed forests at an altitude of approximately 1025 m. All of the types are somewhat teneral.

*Remarks*. The new species resembles *N*. *trifurcatus* Assing, [28] from Sichuan province in external and male sexual characters, but is separated by the wider and apically shallowly concave ventral process of the aedeagus (ventral view).

*Etymology*. The species is named in honor of Xiao-Bin Song, who collected some of the type specimens.

#### *Nazeris congchaoi* Hu & Li sp. n.

urn:lsid:zoobank.org:act:20163A8C-63BA-4D82-8509-2E964B9F0062

*Type material*. Holotype: male: ‘China: Hunan, Yanling County, Taoyuandong National Park, 26°29′14″N, 114°00′42″E, along path in mixed forest, leaf litter, sifted, 770 m, 16.VII.2013, Dai, Peng, Xie leg.’ (SNUC).

*Description*. Body length 6.8 mm; length of forebody 3.7 mm.

**Fig 6:**
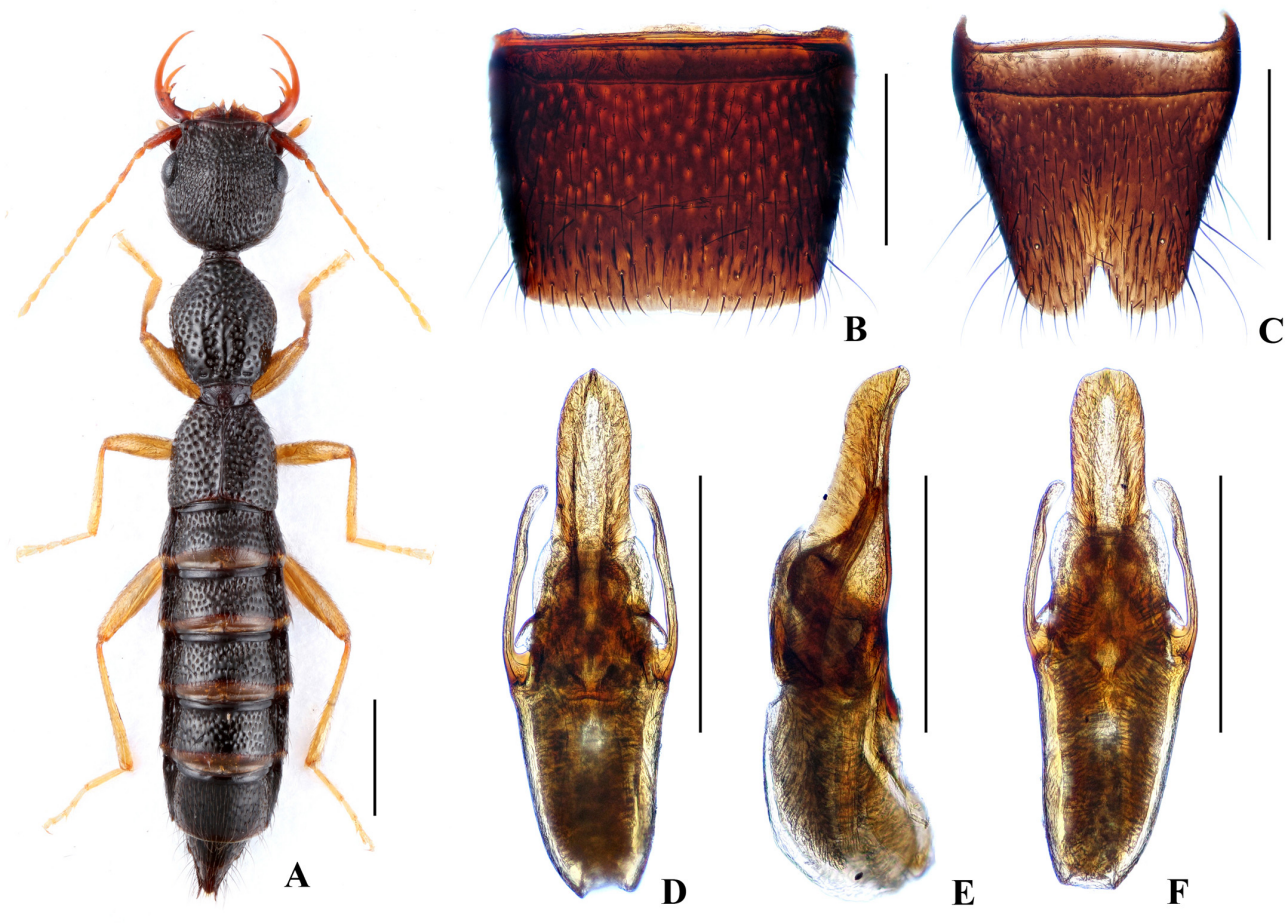
Nazeris congchaoi. (A) habitus. (B) male sternite VII. (C) male sternite VIII. (D) aedeagus, in ventral view. (E) aedeagus, in lateral view. (F) aedeagus, in dorsal view. Scales: A = 1 mm, B–F = 0.5 mm.

Body (Fig 6A) dark brown; antennae legs yellowish brown.

Head as long as wide; punctation very dense, rather coarse, partly confluent, and distinctly umbilicate, interstices without microsculpture; postocular portion 1.65 times as long as eye length.

Pronotum 1.16 times as long as wide, 0.84 times as broad and 0.97 times as long as head; punctation moderately dense and much coarser than that of head; midline with narrow impunctate elevation in posterior half; interstices without microsculpture. Elytra 0.72 times as long as wide, 0.64 times as long and as broad as pronotum; punctation as coarse and as dense as that of pronotum; interstices without microsculpture.

Abdomen with punctation rather dense and coarse on tergites III–VI, slightly less dense but fine on tergites VII–VIII; interstices lacking microsculpture.

*Male*. Sternite VII (Fig 6B) with posterior margin weakly convex at middle. Sternite VIII (Fig 6C) with V-shaped posterior excision. Aedeagus (Fig 6D–6F) weakly sclerotized; ventral process long, slightly widened in basal half in ventral view, curved ventrad near apex in lateral view, with pair of small triangular basal laminae ventrally; dorso-lateral apophyses slender, slightly curved in ventral view, not reaching apex of ventral process.

*Distribution and habitat data*. The type locality is situated to the south of Yanling County, in eastern Hunan. The specimens were collected by sifting leaf litter in a mixed forest at an altitude of 770 m, together with *N*. *pengzhongi*.

*Remarks*. The new species is distinguished from other similarly large *Nazeris* species by the shape of the aedeagus, particularly the long ventral process and the slender dorso-lateral apophyses.

*Etymology*. The species is named in honor of Cong-Chao Dai, one of the collectors of the type material of the new species.

#### *Nazeris nannani* Hu & Li sp. n.

urn:lsid:zoobank.org:act:4AE57ED3-4B2D-423F-9015-BE571FF35C4D

*Type material*. Holotype: male: ‘China: Hunan, Liuyang City, Dawei Shan National Park, 28°25′37″N, 114°07′43″E, along path in mixed forest, leaf litter, sifted, 1430 m, 21.VII.2013, Dai, Peng, Xie leg.’ (SNUC). Paratypes: 1 male: same data as holotype.

*Description*. Body length 5.7–5.8 mm; length of forebody 3.1–3.2 mm.

**Fig 7:**
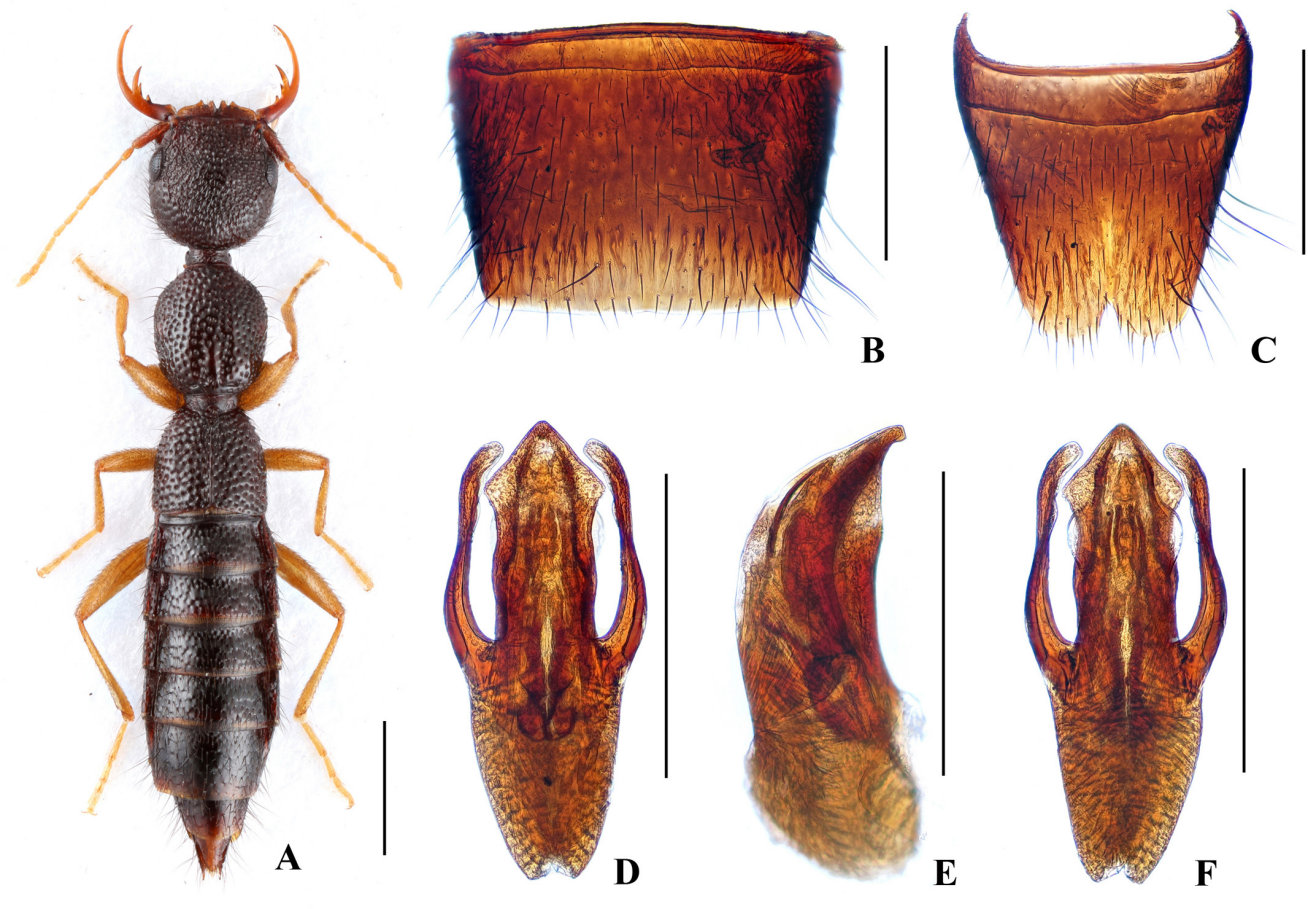
Nazeris nannani. (A) habitus. (B) male sternite VII. (C) male sternite VIII. (D) aedeagus, in ventral view. (E) aedeagus, in lateral view. (F) aedeagus, in dorsal view. Scales: A = 1 mm, B–F = 0.5 mm.

Body (Fig 7A) dark brown; antennae and legs yellowish brown.

Head as long as wide; punctation very dense, rather coarse, partly confluent, and distinctly umbilicate, interstices without microsculpture; postocular portion 1.84 times as long as eye length.

Pronotum 1.12 times as long as wide, 0.88 times as broad and as long as head; punctation moderately dense and much coarser than that of head; midline with narrow impunctate elevation in posterior half; interstices without microsculpture. Elytra 0.67 times as long as wide, 0.61 times as long and as broad as pronotum; punctation as coarse and as dense as that of pronotum; interstices without microsculpture.

Abdomen with punctation dense and moderately coarse on tergites III–V, sparse and fine on tergites VI–VIII; interstices lacking microsculpture.

*Male*. Sternite VII (Fig 7B) with posterior margin weakly concave at middle. Sternite VIII (Fig 7C) with V-shaped posterior excision. Aedeagus (Fig 7D–7F) well sclerotized; ventral process triangularly widened in apical third in ventral view, curved ventrally in lateral view; dorso-lateral apophyses slender, constricted in the middle, slightly curved in ventral view, not reaching apex of ventral process.

*Distribution and habitat data*. The species is known only from Dawei Shan in northeastern Hunan. The specimens were collected by sifting decaying leaf litter in mixed forests at an altitude of 1430 mm, together with *N*. *divisus* and *N*. *daweishanus*.

*Remarks*. The new species resembles *N*. *congchaoi* in external characters and male sternites, but is separated by smaller body size, by sparser and finer abdominal punctation, and by the shorter, apically triangularly widened ventral process.

*Etymology*. The species is named in honor of Nan-Nan Xie, who collected one of the type specimens of the new species.

#### *Nazeris rufus* Hu & Li sp. n.

urn:lsid:zoobank.org:act:F16BF401-FBEB-464F-ADD4-DB6B3EF6D51C

*Type material*. Holotype: male: ‘China: Hunan, Pingjiang Co., Mufushan National Park, 28°59′18″N, 113°49′33″E, along path in mixed forest, leaf litter, sifted, 1550 m, 24.VII.2013, Dai, Peng, Xie leg.’ (SNUC). Paratypes: 11 males, 15 females: same data as holotype.

*Description*. Body length 4.3–4.6 mm; length of forebody 2.3–2.6 mm.

**Fig 8:**
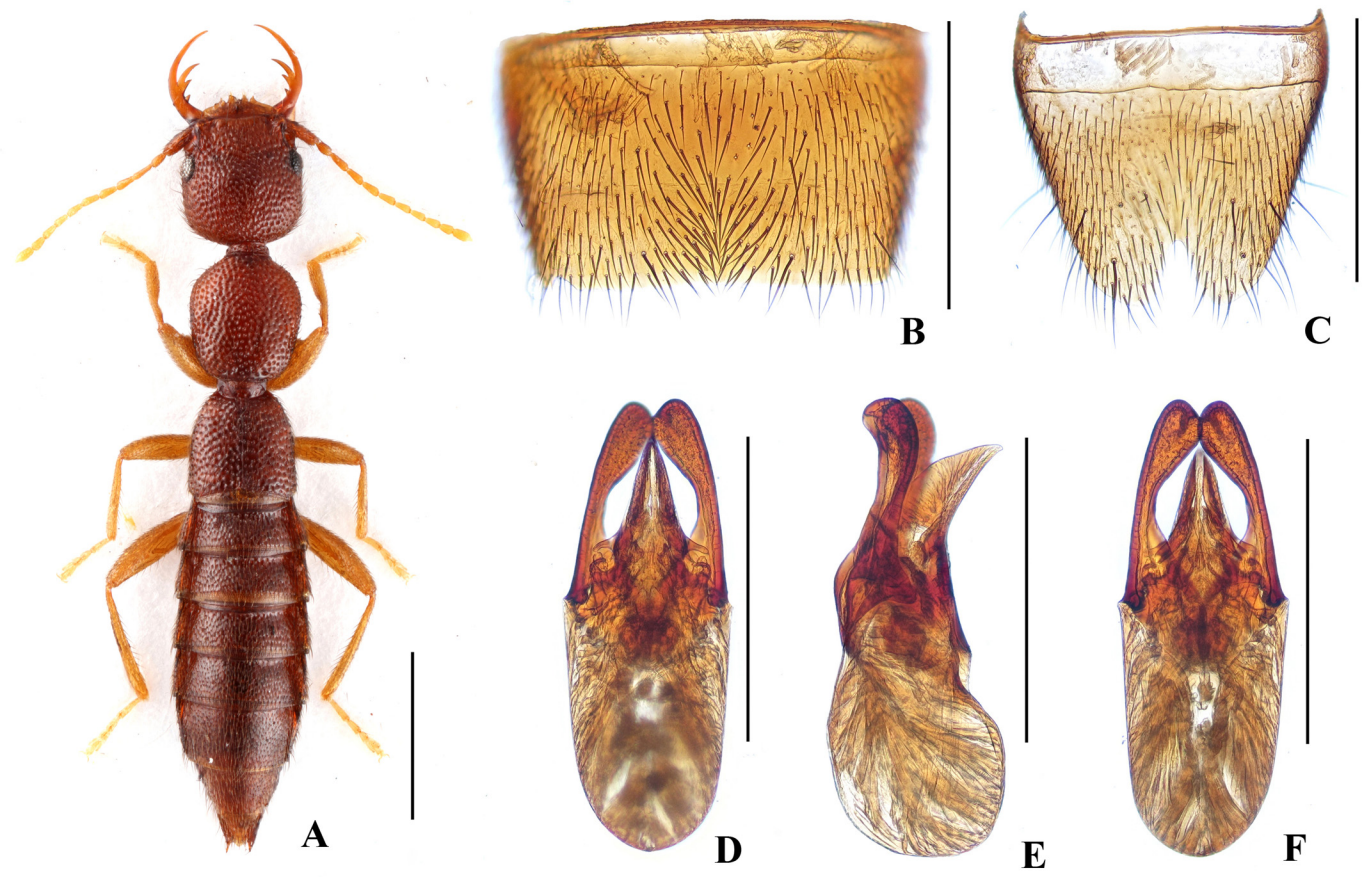
Nazeris rufus. (A) habitus. (B) male sternite VII. (C) male sternite VIII. (D) aedeagus, in ventral view. (E) aedeagus, in lateral view. (F) aedeagus, in dorsal view. Scales: A = 1 mm, B–F = 0.5 mm.

Body (Fig 8A) reddish brown; antennae yellow; legs yellowish brown.

Head longer than wide (length/width = 1.04); punctation very dense, rather coarse, not confluent, and non-umbilicate, interstices without microsculpture; postocular portion 2.25 times as long as eye length.

Pronotum 1.14 times as long as wide, 0.92 times as broad and as long as head; punctation similar to that of head; midline with very narrow or without impunctate elevation in posterior half; interstices without microsculpture. Elytra 0.67 times as long as wide, 0.58 times as long and as broad as pronotum; punctation as coarse and as dense as that of pronotum; interstices without microsculpture.

Abdomen with punctation dense and coarse on tergites III–IV, less dense and finer on tergite V–VI, sparse and fine on tergites VII–VIII; interstices with fine microsculpture.

*Male*. Sternite VII (Fig 8B) with posterior margin indistinctly concave at middle. Sternite VIII (Fig 8C) with V-shaped posterior excision. Aedeagus (Fig 8D–8F) well sclerotized; ventral process short, gradually narrowed apically and with acute apex in ventral view, with pair of small finger-like basal laminae ventrally; dorso-lateral apophyses stout, distinctly thickened apically and slightly curved in ventral view, curved dorsally in lateral view, extending beyond apex of ventral process.

*Distribution and habitat data*. The species is known only from a single locality in northeastern Hunan. The specimens were collected by sifting leaf litter in a mixed forest at an altitude of 1550 m.

*Remarks*. The new species resembles *N*. *sadanarii* Hu & Li, [22] from Anhui province in general appearance especially the tergite microsculpture of abdomen and aedeagal characters, but is distinguished by the relatively broader elytra (as wide as pronotum) and by the apically acute ventral process of the aedeagus.

*Etymology*. The specific epithet (Latin, adjective: red) alludes to the reddish body.

#### *Nazeris ziweii* Hu & Li sp. n.

urn:lsid:zoobank.org:act:EB6A574B-1595-4034-8928-3F4DF4066C78

*Type material*. Holotype: male: ‘China: W. Jiangxi Province, Luxi County, Yangshimu, 27°33′38″N, 114°14′35″E, mixed forest, leaf litter, wood, sifted & beating, ca. 1580 m, 25.X.2013, Peng, Shen & Yan.’ (SNUC). Paratypes: 2 males, 5 females: ‘China: Jiangxi, Pingxiang City, Wugong Shan National Park, 27°27′26″, 114°10′12″E, cableway station to Huiyin Gu, bamboo & pine leaf, sifted, 1500–1750 m, 21.VII.2013, Song, Yin, Yu’.

*Description*. Body length 6.0–6.5 mm; length of forebody 2.6–3.1 mm.

**Fig 9:**
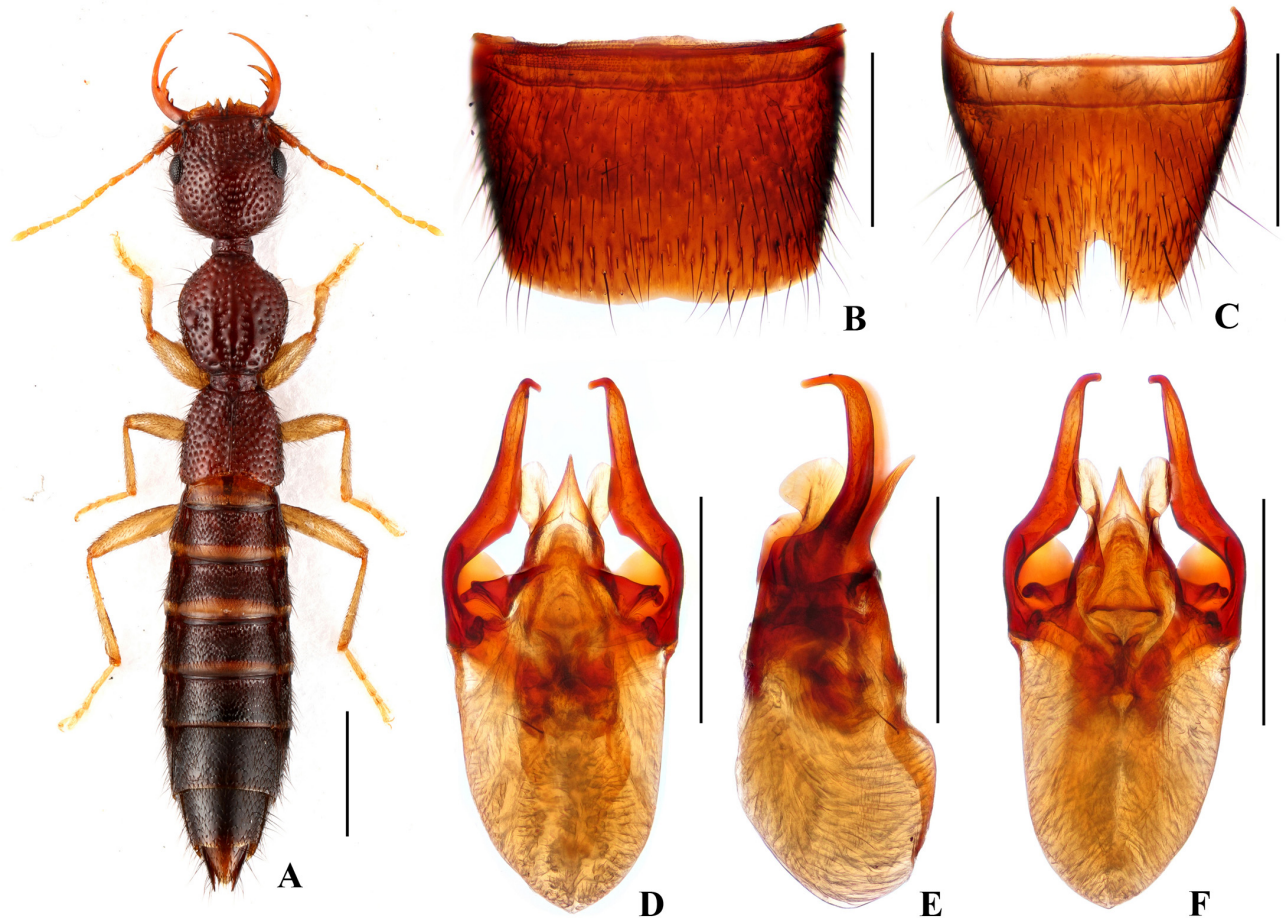
Nazeris ziweii. (A) habitus. (B) male sternite VII. (C) male sternite VIII. (D) aedeagus, in ventral view. (E) aedeagus, in lateral view. (F) aedeagus, in dorsal view. Scales: A = 1 mm, B–F = 0.5 mm.

Body (Fig 9A) dark brown, somewhat reddish; antennae and legs yellowish brown.

Head as long as wide; punctation moderately dense and coarse, not confluent, and non-umbilicate, interstices without microsculpture; postocular portion 1.72 times as long as eye length.

Pronotum 1.13 times as long as wide, 0.93 times as broad and 1.04 times as long as head; punctation slightly finer than that of head; midline with moderately narrow impunctate elevation in posterior half; lateral portions with irregular longitudinal glossy callosities; interstices without microsculpture. Elytra 0.67 times as long as wide, 0.56 times as long and 0.96 times as broad as pronotum; punctation slightly denser but less coarse than that of pronotum; interstices without microsculpture.

Abdomen with punctation dense and coarse on tergites III–V, less dense and finer on tergite VI, sparse and fine on tergites VII–VIII; interstices lacking microsculpture.

*Male*. Sternite VII (Fig 9B) with posterior margin weakly concave at middle. Sternite VIII (Fig 9C) with U-shaped posterior excision. Aedeagus (Fig 9D–9F) weakly sclerotized; ventral process short, gradually narrowed apically and with acute apex in ventral view, with pair of oval laminae in apical half on dorsal side and triangular laminae near base on ventral side; dorso-lateral apophyses long, with widened middle part and sharp apex, distinctly curved in basal half in ventral view, curved dorsad near apex in lateral view, extending far beyond apex of ventral process.

*Distribution and habitat data*. The species was found in two adjacent localities in the central part of Luoxiao Shan in northwestern Jiangxi. The specimens were collected by sifting mixed leaf litter, bamboo, and pine litter at altitudes of 1500–1750 m, partly together with *N*. *luoxiaoshanus*. Two paratypes are teneral.

*Remarks*. The new species is distinguished from all the known species of *Nazeris* by the distinctive shape of the aedeagus, particularly the short and narrow ventral process with a pair of oval dorsal laminae apically, as well as the long and curved dorso-lateral apophyses.

*Etymology*. The species is named in honor of Zi-Wei Yin, who collected part of the type material of the new species.

#### *Nazeris daweishanus* Hu & Li sp. n.

urn:lsid:zoobank.org:act:691A9C60-13E5-474C-B63E-E1FEDECE4967

*Type material*. Holotype: male: ‘China: Hunan, Liuyang City, Dawei Shan National Park, 28°25′37″N, 114°07′43″E, along path in mixed forest, leaf litter, sifted, 1430 m, 21.VII.2013, Dai, Peng, Xie leg.’ (SNUC). Paratypes: 3 males, 4 females: same data as holotype.

*Description*. Body length 4.5–4.8 mm; length of forebody 2.6–2.8 mm.

**Fig 10:**
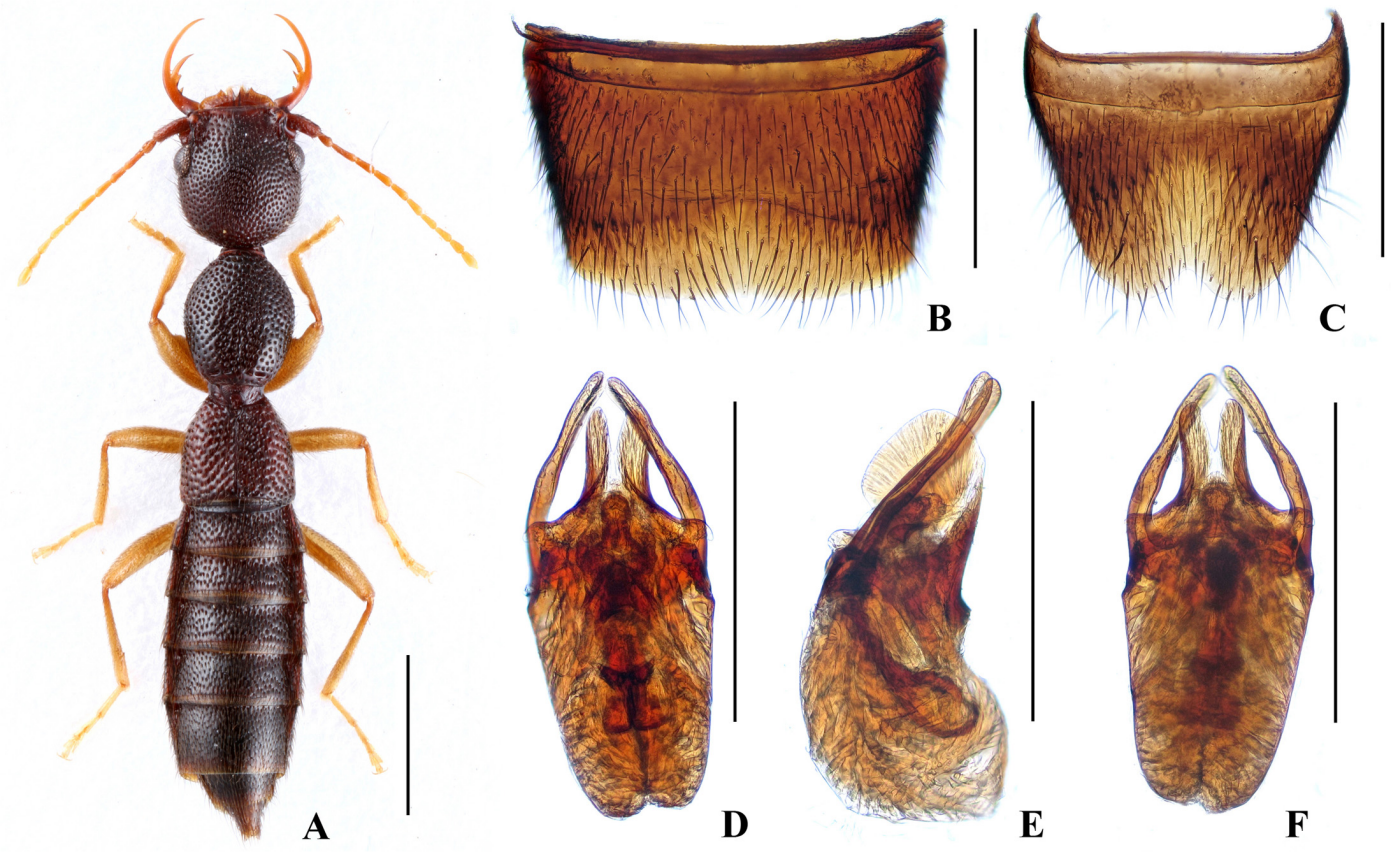
Nazeris daweishanus. (A) habitus. (B) male sternite VII. (C) male sternite VIII. (D) aedeagus, in ventral view. (E) aedeagus, in lateral view. (F) aedeagus, in dorsal view. Scales: A = 1 mm, B–F = 0.5 mm.

Body (Fig 10A) dark brown; antennae and legs yellowish brown.

Head longer than wide (length/width = 1.05); punctation very dense, moderately coarse, not confluent, and non-umbilicate, interstices without microsculpture; postocular portion 2.04 times as long as eye length.

Pronotum 1.20 times as long as wide, 0.84 times as broad and 0.97 times as long as head; punctation dense and slightly coarser than that of head; midline posteriorly with short and very narrow impunctate elevation; lateral portions without distinct impressions or elevations; interstices without microsculpture. Elytra 0.65 times as long as wide, 0.56 times as long and 1.04 times as broad as pronotum; punctation as coarse and as dense as that of pronotum; interstices without microsculpture.

Abdomen with punctation rather dense and coarse on tergites III–V, dense and less coarse on tergite VI, moderately dense and fine on tergites VII–VIII; interstices lacking microsculpture.

*Male*. Sternite VII (Fig 10B) with posterior margin weakly convex at middle. Sternite VIII (Fig 10C) with broadly triangular posterior excision. Aedeagus (Fig 10D–10F) weakly sclerotized; ventral process short, with parallel sides, apically with V-shaped excision in ventral view, with pair of small basal laminae ventrally; dorso-lateral apophyses slender, nearly straight in ventral view or lateral view, extending beyond apex of ventral process.

*Distribution and habitat data*. The species is known only from Dawei Shan in northeastern Hunan. The specimens were collected by sifting decaying leaf litter in mixed forests at an altitude of 1430 m, together with *N*. *nannani* and *N*. *divisus*. Three paratypes are teneral.

*Remarks*. The new species resembles *N*. *cultellatus* Assing, [28] from Shaanxi, Henan and Anhui province in general appearance especially the body size, punctation, and male sexual characters, but is distinguished by the shorter and narrower ventral process of the aedeagus.

*Etymology*. The specific epithet is derived from Dawei Shan, where the species was discovered.

#### *Nazeris prominens* Hu & Li sp. n.

urn:lsid:zoobank.org:act:9BD9B83E-28F2-4342-8F73-A5AC0B228E6A

*Type material*. Holotype: male: ‘China: W. Jiangxi, Yichun City, Mingyueshan National Park, 27°35′32–46″N, 114°17′13″–16′40″E, path in mixed forest, grass, leaf litter, sifted, 1200–1600 m, 12.VII.2013, Song, Yin, Yu leg.’ (SNUC). Paratypes: 3 males, 1 female: same data as holotype; 1 male: ‘China: W. Jiangxi, Yichun City, Mingyueshan, 27°35′13″N, 114°16′53″E, mixed forest, leaf litter, wood, sifted & beating, ca. 1600 m, 22.X.2013, Peng, Shen & Yan’.

*Description*. Body length 5.1–5.8 mm; length of forebody 2.7–2.9 mm.

**Fig 11:**
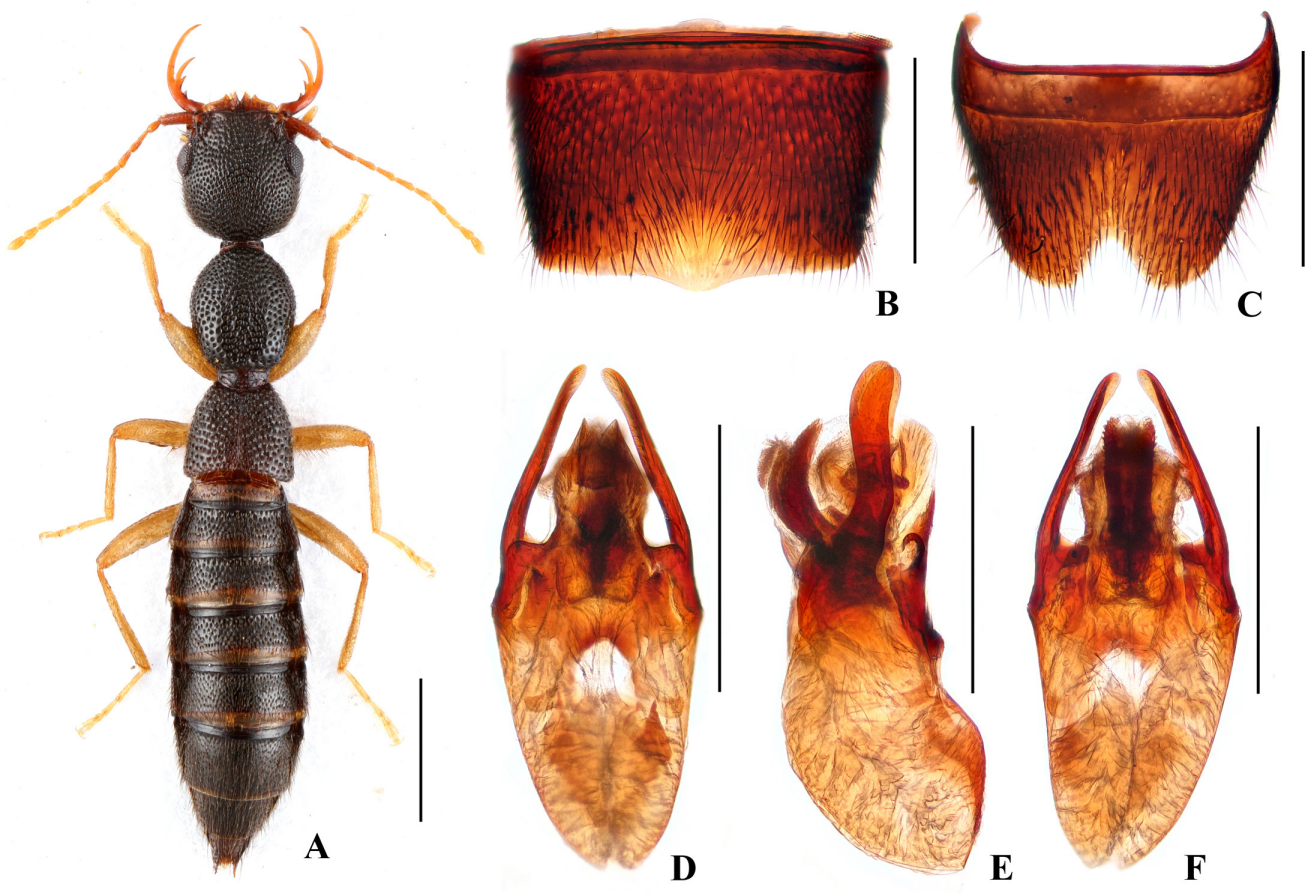
Nazeris prominens. (A) habitus. (B) male sternite VII. (C) male sternite VIII. (D) aedeagus, in ventral view. (E) aedeagus, in lateral view. (F) aedeagus, in dorsal view. Scales: A = 1 mm, B–F = 0.5 mm.

Body (Fig 11A) dark brown; antennae and legs yellowish brown.

Head as long as wide; punctation very dense, moderately coarse, not confluent, and non-umbilicate, interstices without microsculpture; postocular portion 1.63 times as long as eye length.

Pronotum 1.21 times as long as wide, 0.83 times as broad and as long as head; punctation dense and moderately coarser than that of head; midline posteriorly with short and very narrow impunctate elevation; lateral portions without distinct impressions or elevations; interstices without microsculpture. Elytra 0.64 times as long as wide, 0.55 times as long and 1.04 times as broad as pronotum; punctation as coarse and as dense as that of pronotum; interstices without microsculpture.

Abdomen with punctation rather dense and coarse on tergites III–V, dense and less coarse on tergite VI, moderately dense and fine on tergites VII–VIII; interstices lacking microsculpture.

*Male*. Sternite VII (Fig 11B) with posterior margin distinctly prominent at middle. Sternite VIII (Fig 11C) with broadly V-shaped posterior excision. Aedeagus (Fig 11D–11F) well sclerotized; ventral process short, with convex sides and triangularly excised apex in ventral view, with pair of small basal laminae ventrally; dorso-lateral apophyses slender and nearly straight in ventral view, slightly curved dorsad in lateral view, extending beyond apex of ventral process.

*Distribution and habitat data*. The species was found in Mingyue Shan in the central part of Luoxiao Shan in western Jiangxi. The specimens were collected by sifting grass and leaf litter in a mixed forest at altitudes of 1500–1750 m, partly together with *N*. *luoxiaoshanus* or *N*. *inaequalis*. One paratype is teneral.

*Remarks*. The new species resembles *N*. *cultellatus* in general appearance and male sexual characters, but is distinguished by the shape of the male sternite VII (posterior margin distinctly prominent at middle), by the shorter ventral process of the aedeagus, and by the narrower dorso-lateral apophyses.

*Etymology*. The specific epithet (Latin, adjective: prominent) alludes to the prominent middle of the posterior margin of the male sternite VII.

#### *Nazeris zekani* Hu & Li sp. n.

urn:lsid:zoobank.org:act:42ADBBE2-3F5D-4A2B-A1AE-83018B62F59D

*Type material*. Holotype: male: ‘China: Hunan, Liuyang City, Daweishan National Park, 28°25′28″N, 114°04′52″E, along path in mixed forest, leaf litter, sifted, 830 m, 22.VII.2013, Dai, Peng, Xie leg.’ (SNUC). Paratypes: 5 males, 3 females: same data as holotype; 7 males, 5 females: ‘China: W. Jiangxi, Yichun City, Fengxin County, Yue Shan, 28°47′03″N, 115°10′25″E, mixed leaf litter, sifted, 800–900 m, 23.VII.2013, J. Y. Hu & Z. K. Lv leg.’.

*Description*. Body length 4.9–5.1 mm; length of forebody 2.6–2.9 mm.

**Fig 12:**
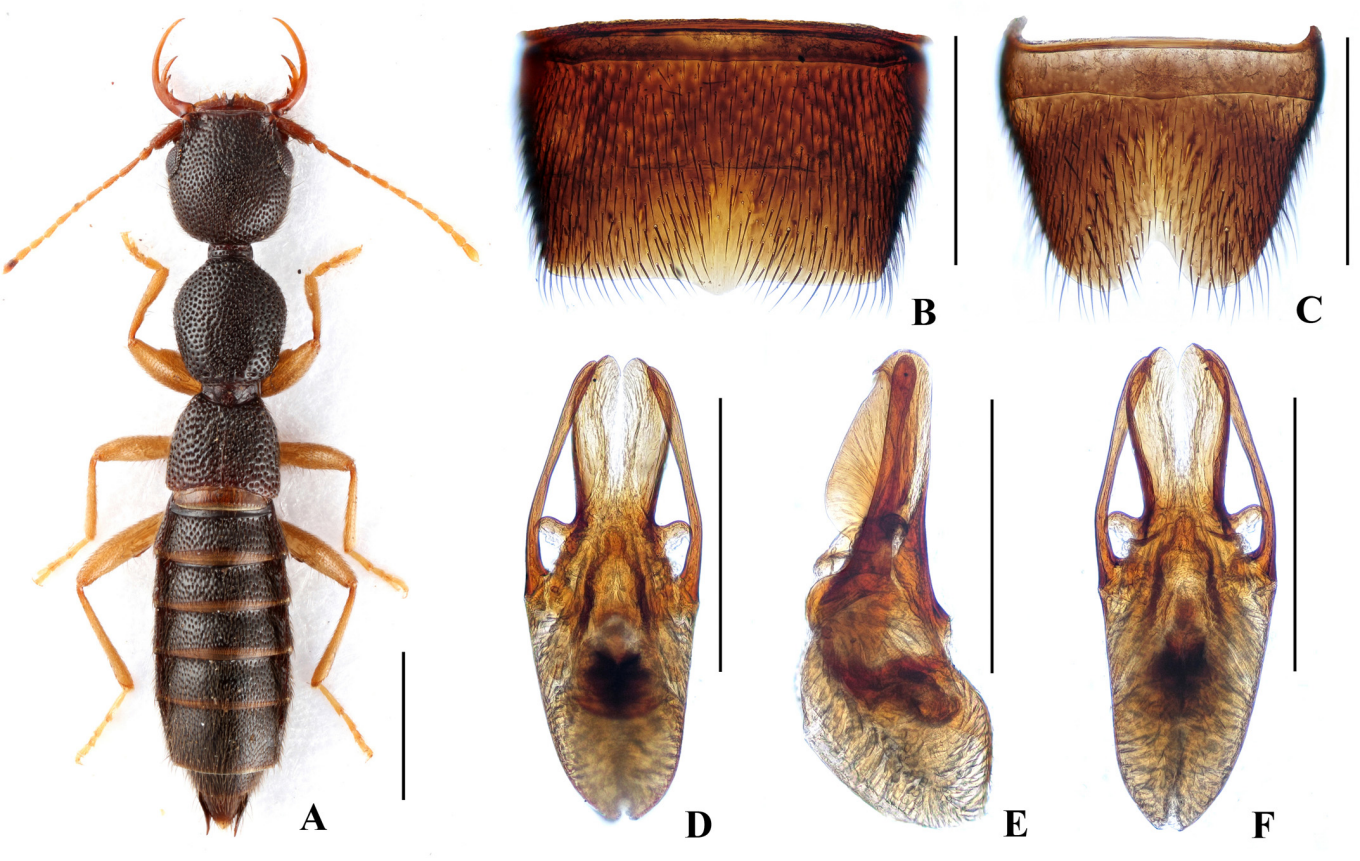
Nazeris zekani. (A) habitus. (B) male sternite VII. (C) male sternite VIII. (D) aedeagus, in ventral view. (E) aedeagus, in lateral view. (F) aedeagus, in dorsal view. Scales: A = 1 mm, B–F = 0.5 mm.

Body (Fig 12A) dark brown; antennae and legs yellowish brown.

Head longer than wide (length/width = 1.03); punctation very dense, moderately coarse, not confluent, and non-umbilicate, interstices without microsculpture; postocular portion 1.45 times as long as eye length.

Pronotum 1.11 times as long as wide, 0.88 times as broad and 0.95 times as long as head; punctation dense and slightly coarser than that of head; midline posteriorly with short and very narrow impunctate elevation; lateral portions without distinct impressions or elevations; interstices without microsculpture. Elytra 0.62 times as long as wide, 0.54 times as long and as broad as pronotum; punctation as coarse and as dense as that of pronotum; interstices without microsculpture.

Abdomen with punctation rather dense and coarse on tergites III–V, dense and less coarse on tergite VI, moderately dense and fine on tergites VII–VIII; interstices lacking microsculpture.

*Male*. Sternite VII (Fig 2) with posterior margin distinctly prominent at middle. Sternite VIII (Fig 3) with V-shaped posterior excision. Aedeagus (Figs 4–6) weakly sclerotized; ventral process moderately long, constricted in basal third, with triangularly excised apex in ventral view, with pair of small basal laminae ventrally; dorso-lateral apophyses slender, weakly curved in ventral view, nearly straight in lateral view, not reaching apex of ventral process.

*Distribution and habitat data*. The species was found in two localities in the northern part of Luoxiao Shan in northeastern Hunan and northwestern Jiangxi. The specimens were collected by sifting mixed leaf litter at altitudes between 800 and 900 m, partly together with *N*. *divisus*. Three paratypes are teneral.

*Remarks*. The new species resembles *N*. *prominens* in body size, punctation and male sternites, but can be distinguished by the different aedeagal structure: ventral process much longer and dorso-lateral apophyses more slender.

*Etymology*. The species is named in honor of Ze-Kan Lv, who collected some of the type specimens of the new species.
